# Suboptimal Criterion Learning in Static and Dynamic Environments

**DOI:** 10.1371/journal.pcbi.1005304

**Published:** 2017-01-03

**Authors:** Elyse H. Norton, Stephen M. Fleming, Nathaniel D. Daw, Michael S. Landy

**Affiliations:** 1 Department of Psychology, New York University, New York, New York, United States of America; 2 Wellcome Trust Centre for Neuroimaging, University College London, London, United Kingdom; 3 Department of Psychology, Princeton University, Princeton, New Jersey, United States of America; 4 Princeton Neuroscience Institute, Princeton University, Princeton, New Jersey, United States of America; 5 Center for Neural Science, New York University, New York, New York, United States of America; University of Birmingham, UNITED KINGDOM

## Abstract

Humans often make decisions based on uncertain sensory information. Signal detection theory (SDT) describes detection and discrimination decisions as a comparison of stimulus “strength” to a fixed decision criterion. However, recent research suggests that current responses depend on the recent history of stimuli and previous responses, suggesting that the decision criterion is updated trial-by-trial. The mechanisms underpinning criterion setting remain unknown. Here, we examine how observers learn to set a decision criterion in an orientation-discrimination task under both static and dynamic conditions. To investigate mechanisms underlying trial-by-trial criterion placement, we introduce a novel task in which participants explicitly set the criterion, and compare it to a more traditional discrimination task, allowing us to model this explicit indication of criterion dynamics. In each task, stimuli were ellipses with principal orientations drawn from two categories: Gaussian distributions with different means and equal variance. In the covert-criterion task, observers categorized a displayed ellipse. In the overt-criterion task, observers adjusted the orientation of a line that served as the discrimination criterion for a subsequently presented ellipse. We compared performance to the ideal Bayesian learner and several suboptimal models that varied in both computational and memory demands. Under static and dynamic conditions, we found that, in both tasks, observers used suboptimal learning rules. In most conditions, a model in which the recent history of past samples determines a belief about category means fit the data best for most observers and on average. Our results reveal dynamic adjustment of discrimination criterion, even after prolonged training, and indicate how decision criteria are updated over time.

## Author Summary

Understanding how humans make decisions based on uncertain sensory information is crucial to understanding how humans interpret and act on the world. Signal detection theory models discrimination and detection decisions as a comparison of “stimulus strength” to a fixed criterion. In a world that is constantly changing a static criterion makes little sense. We investigate this as a problem of learning: How is the decision criterion set when various aspects of the context are unknown (e.g., category means and variances)? We examine criterion learning in both static and dynamic environments. In addition to a more traditional discrimination task in which the criterion is a theoretical construct and unobservable, we use a novel task in which participants must explicitly set the criterion before being shown the stimulus. We show that independent of environment and task, observers dynamically update the decision criterion, even after prolonged training in a static environment. Our results provide evidence against an assumption of stability and have implications for how psychophysical data are analyzed and interpreted and how humans make discrimination decisions under uncertainty.

## Introduction

Understanding how humans make decisions based on uncertain sensory information is crucial to understanding how humans interpret and act on the world. For over 60 years, signal detection theory has been used to analyze detection and discrimination tasks [1]. Typically, sensory data are assumed to be Gaussian with equal variances but different means for signal-absent and signal-present trials. To decide, the observer compares the noisy sensory data to a fixed decision criterion. Performance is summarized by *d*′ (discriminability) and *c* (decision criterion) based on measured hit and false-alarm rates. Standard analysis assumes stable performance (all parameters fixed) and observer knowledge of the means, variance, prior probabilities and payoff matrix [1,2,3,4,5,6,7].

The assumption of stable performance is problematic for two reasons. (1) Observers may learn about the environment and use that information to set the decision criterion. (2) The environment may not be stable or the observer may not believe that the environment is stable. To circumvent these problems, researchers include training sessions, fix the environmental parameters (e.g., priors, payoffs) within blocks, and treat learning effects as additional noise (i.e., its “variance” can simply be added to those of internal and/or external noise in the experiment). However, research investigating history effects in psychophysical tasks has shown that an observer’s current decision is affected by multiple aspects of the stimulus history (e.g., recent decisions, stimulus intervals, trial type, etc.). These effects occur even when the environment is stable, the stimulus presentation is random, and observers are well trained [8,9,10,11,12,13]. Observers behave as if the environment is dynamic and, as a result, measures of discriminability and sensitivity are biased and the confidence intervals computed for the best fitting parameters of the psychometric function are too narrow [14]. While assuming instability in a static world is suboptimal, in a world that is constantly changing a fixed criterion makes little sense.

To optimize decisions in dynamic environments, observers must update decision criteria in response to changes in the world by adapting to the value and uncertainty of sensory information. Humans respond appropriately to changes in visual and motor uncertainty [15,16,17,18,19,20]. Observers adjust the decision criterion when uncertainty is varied randomly from trial to trial [18]. If the location of visual feedback for a reach is perturbed dynamically over trials, participants track this random walk near-optimally [15]. Landy and colleagues [17] demonstrated that participants tracked discrete changes in the variance of a visual perturbation. Summerfield and colleagues [19] investigated a visual discrimination task in which participants categorized gratings with orientations drawn from two overlapping distributions. Means and variances were updated randomly with different levels of volatility. Participants’ performance changed as a function of volatility.

While the above studies examine the dynamics of decision-making, they only provide indirect evidence of criterion shifts. Many of these studies observed changes in decisions and response time, but few studies have examined how trial history specifically affects decision criteria and what is the underlying mechanism responsible for learning and updating the decision criterion. Lages and Treisman [13] describe the dynamics of criterion setting and updates of priors based on previous stimulus samples and responses applied to tasks with no experimenter feedback, so that the criterion drifts to the mean of previously experienced stimuli. Summerfield and colleagues [19] consider a discrimination task with feedback in which the categories and their associated uncertainty can change several times per block of trials. They compare several suboptimal models, all of which predict the choice probability by probability matching.

Here, we investigate how humans learn to set and update criteria for perceptual decisions in both static and dynamic environments. To examine the underlying mechanisms of criterion learning, we take a quantitative approach and compare models of how a decision criterion is set as a function of recently experienced stimuli and feedback. Observers completed two different experimental tasks. One task was the typical discrimination task, in which the observer’s criterion is unobservable. We introduce a novel overt-criterion task, in which the decision criterion is set explicitly by the observer. This allows us to measure and model the setting of the decision criterion directly. We used the overt-criterion task, which has greater statistical power due to the richer dataset, to develop and test models of how the criterion is updated in standard discrimination experiments under uncertainty. In contrast to the models investigated by Summerfield and colleagues [19], we directly measure the criterion, and include parameters for sensory noise and predict a specific response based on the noisy stimulus information and a model of criterion update. While observers converged to the optimal criterion over many trials when conditions were static and followed dynamic changes in the category means, we found that, in both tasks, the majority of observers used suboptimal learning rules. Our results reveal dynamic adjustment of a discrimination criterion, even after prolonged training in a static environment.

## Results

### Experiment 1

**Fig 1:**
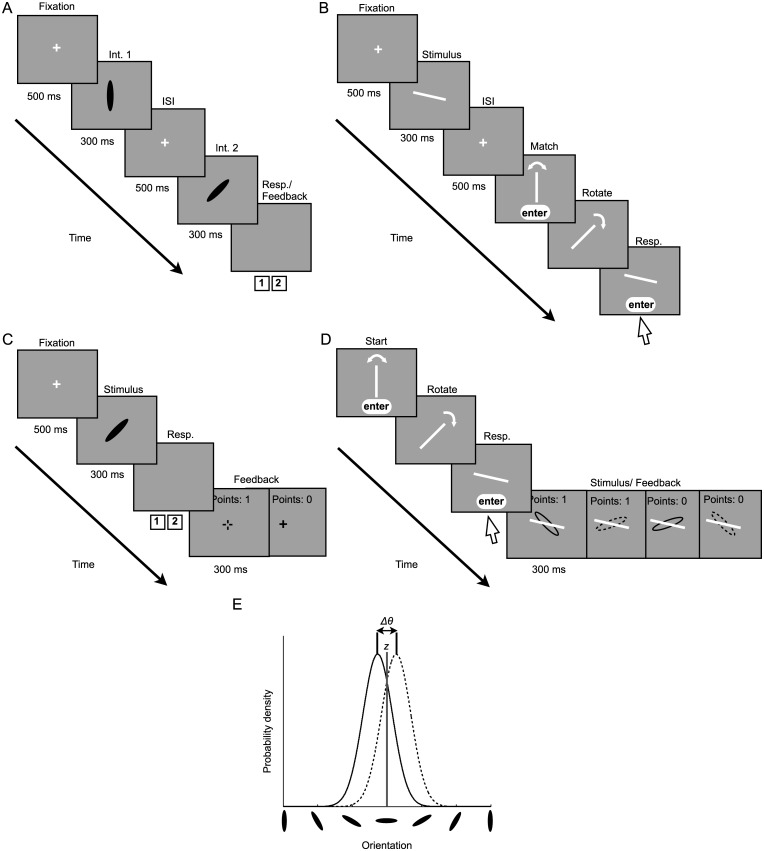
Example trial sequences and category distributions. **A**) The orientation-discrimination task. The observer’s task was to choose the interval containing the more clockwise ellipse. **B**) The orientation-matching task. The observer had to best match the orientation of a line that was displayed on the screen. In the experiment, the lines were yellow on a gray background. **C**) The covert-criterion task. The observers categorized ellipses as belonging to the category *A* or category *B* distribution with the 1 and 2 keys, respectively. The subsequent fixation cross indicated the correct category (green for A, displayed here as solid; red for B, displayed as dashed). **D**) The overt-criterion task. On each trial, observers adjusted the orientation of a yellow line (shown here as white) that served as the discrimination criterion for the subsequently presented ellipse. Feedback ellipses were green and red, here displayed as solid and dashed. In the covert- and overt-criterion tasks, stimuli were ellipses with principal orientations drawn from two categories *A* and *B*: Gaussian distributions with different means and equal variance. **E**) Category distributions. The solid curve represents the distribution underlying stimuli belonging to category *A* and the dashed curve represents the distribution underlying the stimuli belonging to category *B*. The distance between the two distributions (Δ*θ*) was set such that difficulty was equated across observers (*d*′ = 1). The optimal criterion (*z*) is represented by the solid gray line and falls directly between the two category means. The means of the distributions were chosen randomly at the beginning of each block, remained constant throughout the block in Expt. 1, and were updated on every trial via a random walk in Expt. 2.

**Fig 2:**
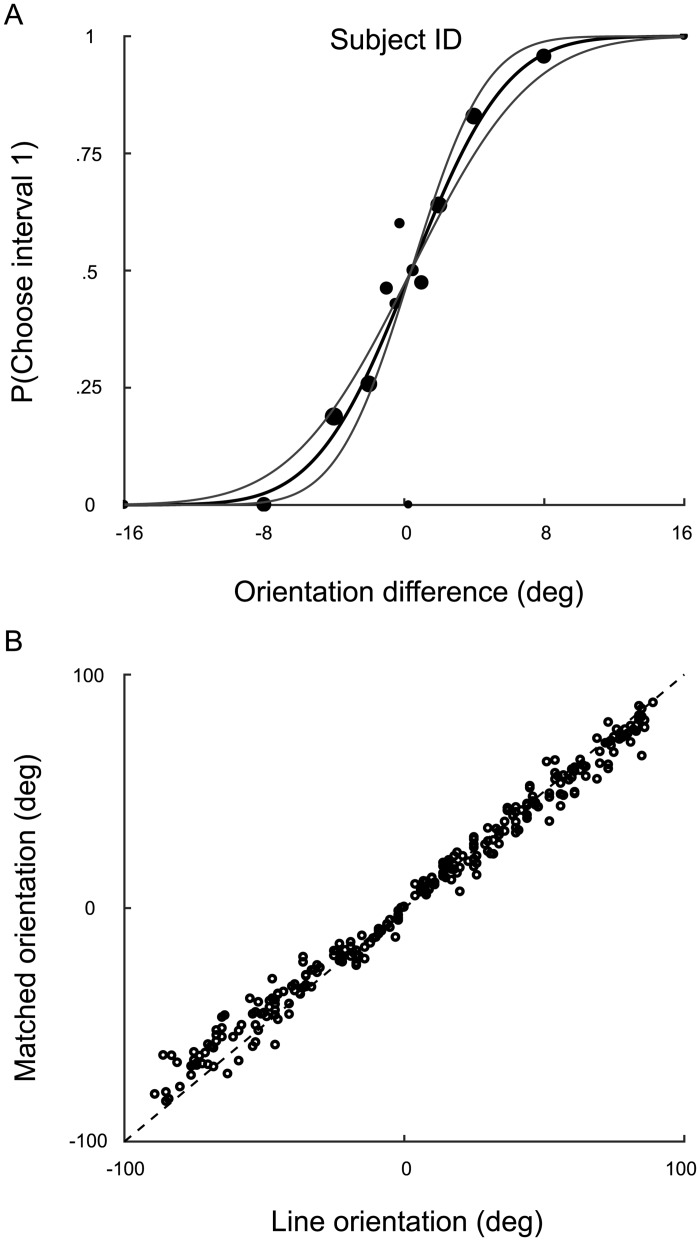
Discrimination and matching data. **A**) A psychometric function for a representative observer in the orientation-discrimination task. Data points: raw data. Circle area is proportional to the number of trials completed at the corresponding orientation difference (Δ*θ*). A cumulative normal distribution was fit to the data (solid black line). The gray curves represent a 95% confidence interval on the slope parameter. **B**) One observer’s raw data from the orientation-matching task. The orientation of the matched line is shown as a function of the orientation of the displayed line. The identity line indicates a perfect match.

**Fig 3:**
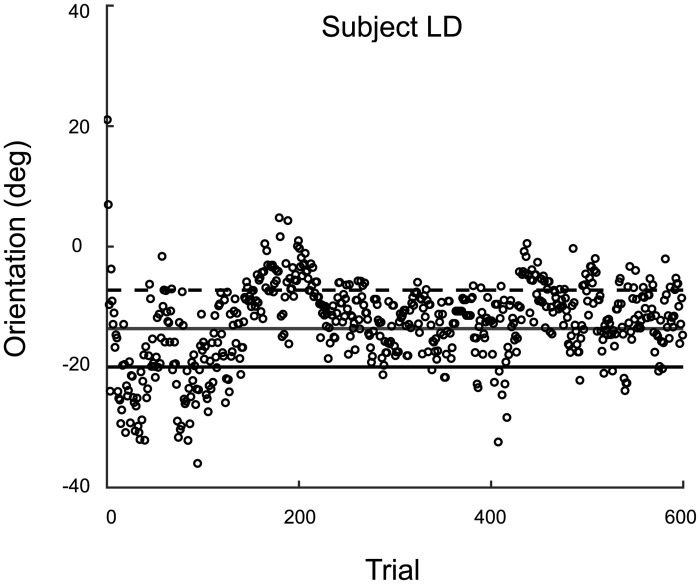
Overt-criterion data for a representative observer in Expt. 1. Data points: Criterion placement on each trial. Lines, The mean orientation of the category *A* and *B* distributions (solid and dashed, respectively) and the optimal observer’s criterion (solid gray).

All observers completed three tasks: (1) An orientation-discrimination task in which discrimination thresholds were measured and used to equate the difficulty of the covert- and overt-criterion tasks across observers (Fig 1*A*), (2) A covert-criterion task in which observers categorized an ellipse as belonging to category *A* or *B* (Fig 1*C*), and (3) An overt-criterion task in which observers explicitly indicated their criterion on each trial prior to the presentation of a category *A* or *B* ellipse (Fig 1*D*). Additionally, 8 out of 10 observers completed an orientation-matching task in which adjustment noise was measured (Fig 1*B*). Categories in the covert- and overt-criterion tasks were Gaussian distributions with different mean orientations and equal variance (Fig 1*E*; see Methods).

#### Estimating sensory uncertainty

In the orientation-discrimination task, we quantified sensory uncertainty (*σ*_*v*_) for each observer by determining the just noticeable difference in orientation between two sequentially presented ellipses. We calculated the probability of choosing interval one as a function of the orientation difference between the ellipses and fit a cumulative normal distribution to the data using a maximum-likelihood criterion with parameters *μ*, *σ*, and *λ* (the mean, SD, and lapse rate). We define threshold as the underlying measurement SD *σ*_*v*_ (correcting for the 2IFC task by dividing by $\sqrt{2}$). A 95% confidence interval for *σ*_*v*_ was obtained by a parametric bootstrap method in which the estimated parameters were used to generate 10,000 experimental simulations, re-fit, and the 2.5 and 97.5 percentiles were calculated. Fig 2*A* shows a representative psychometric function. On average, *σ*_*v*_ = 6.0° with individual observer values ranging from 3.0° to 10.6°.

#### Estimating adjustment uncertainty

In the orientation-matching task, we quantified adjustment uncertainty (*σ*_*a*_) for the eight observers who completed the matching task. For each observer, we calculated the standard deviation ${\bar{\sigma }}_{a}$ of setting errors. Raw data for a representative observer are shown in Fig 2*B*. On average, ${\bar{\sigma }}_{a}=7.2^{{}^{\circ}}$ with individual observers ranging from 6.0 to 9.1°.

#### Raw overt-criterion data

In the overt-criterion block, to determine whether or not observers learned the optimal criterion we looked at trial-by-trial criterion placement and deviation from the optimal criterion. The raw data for a sample observer are plotted in Fig 3. The average root-mean-square error from the optimal criteria across observers was 6.9° with individual observers’ error ranging from 4.1 to 11.3°.

#### Response to recently experienced stimuli

A regression analysis was performed to examine the dependence of binary decisions in the covert-criterion task and criterion placement in the overt-criterion task on an observer’s recent experience. This allowed us to determine how observers combined information from multiple trials into an estimate of the category means without assuming an underlying parametric model. We conducted “lagged regressions” on data from each task in which we included the orientations of the nine most recently experienced ellipses from each category as regressors.

**Fig 4:**
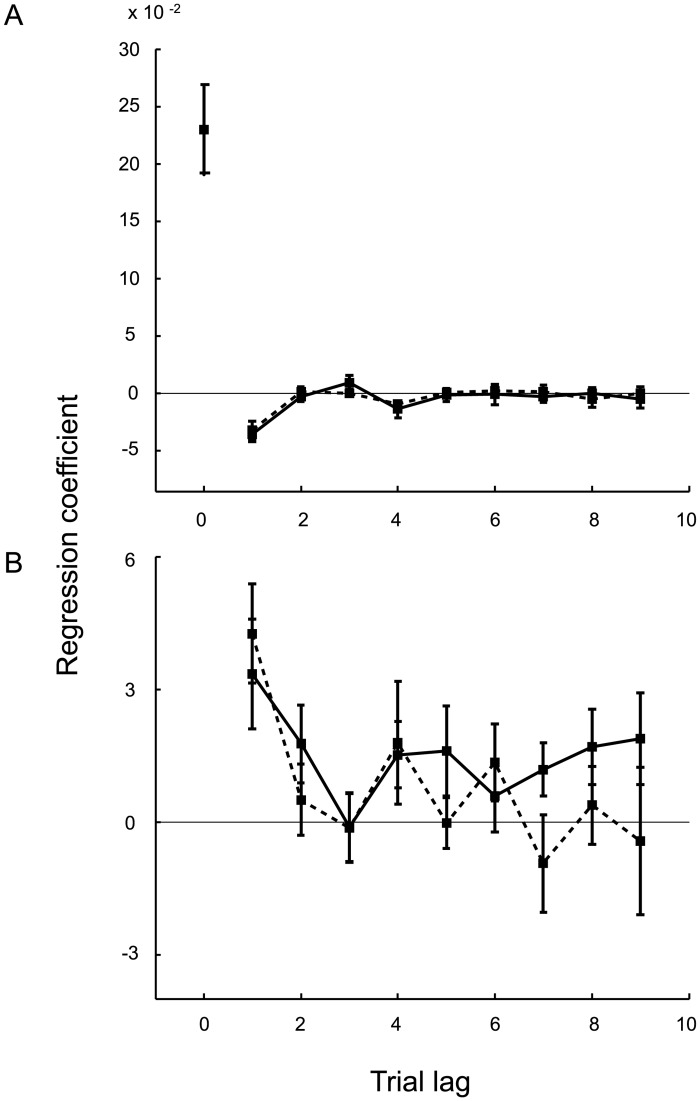
Lagged regression for the static condition (Expt. 1). **A**) Covert-criterion task: Results of a logistic regression predicting the binary decision of each trial as a combination of the orientations of the current ellipse and the previous nine ellipses in each category. The solid and dashed lines represent the group average beta weights ±SE for the ellipses belonging to category *A* and category *B*, respectively. **B**) Overt-criterion task: Results of a linear regression predicting the criterion placement on each trial as a combination of the orientations of the previous nine ellipses in each category. Again, the solid and dashed lines represent the group average beta weights ±SE for the ellipses belonging to category *A* and category *B*, respectively.

The overall trend is noisy but appears to weight recent ellipses more heavily than those further in the past (Fig 4). Specifically, for the covert-criterion task (Fig 4*A*) we find a positive weight for the current stimulus and a negative weight for the previous trial but at a fraction of the value ($\frac{\beta_{A,\;n-1}}{\beta_{A,\;n}}=-.16$ and $\frac{\beta_{B,\;n-1}}{\beta_{B,\;n}}=-.14$). The weights for the remaining seven trials converge to zero (i.e., a shape that does not rule out an exponential form from lag one to lag nine). This pattern of results is consistent with the idea that the decision on the current trial is simply the difference between the current stimulus and the criterion, which is determined by taking a weighted average of the previous stimuli from both categories (i.e., the criterion is the average of the two category means, Eq 4). For the overt-criterion task (Fig 4*B*), the shape for both categories also does not rule out an exponential form, again suggesting that the current criterion setting is a weighted average of the previous stimuli from the two categories. However, few of the beta values were significantly nonzero. In the analysis of the covert-criterion data, the average influence of category *A* ellipses differed significantly from zero for one of the nine lags and the category *B* ellipses differed significantly from zero for two of the nine lags (*p* < 0.05). In the overt-criterion block, the average influence of category *A* and *B* ellipses each differed significantly from zero for one of the nine lags (*p* < 0.05).

#### Model comparison

We compared five models of criterion learning that varied in both computational and memory demands (see Methods). (1) The ideal Bayesian observer computes the posterior probabilities of an ellipse belonging to both categories and integrates across the unknown category parameters. (2) The Bayesian model-selection observer estimates the means of each category by averaging all previously experienced stimuli. (3) The exponentially weighted moving-average observer estimates the means of each category using a weighted average where more weight is given to recently experienced stimuli. (4) The reinforcement-learning observer only updates the criterion when receiving negative feedback. The criterion is then moved by a fixed fraction of the difference between its current value and the value of the stimulus. (5) The limited-memory observer approximates the mean for each category by using the most recently experienced stimulus from that category.

**Fig 5:**
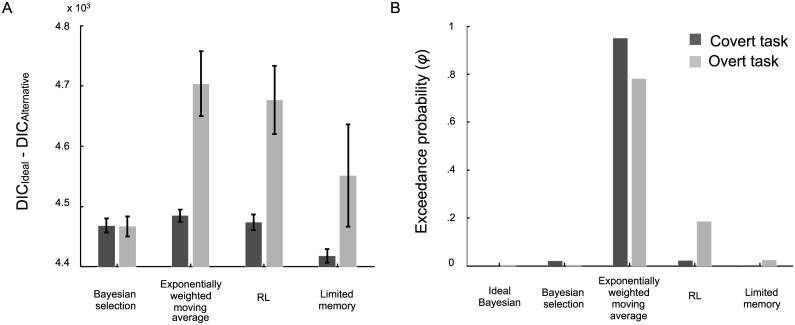
Model comparison results for the covert- (dark gray) and overt-criterion (light gray) tasks in Expt. 1. **A**) The bar graph depicts the relative DIC scores (i.e., DIC difference between the ideal Bayesian model and the suboptimal models) averaged across observers ±SE. Larger values indicate a better fit. **B**) To summarize the results from the group level analysis we computed exceedance probabilities for each model in each task. A model’s exceedance probability tells us how much more likely that model is compared to the alternatives, given the group data.

To obtain a quantitative measure of model fit, we computed DIC scores for each combination of task, observer and model. Tables 1 and 2 contain the median DIC scores for each observer and model in the covert-criterion task and the overt-criterion task, respectively. The best fitting model(s) for each observer is indicated. We allowed for ties, so occasionally multiple models were the best fitting and are indicated as such. Models tied if their DIC scores were within seven of the lowest-scoring model. Fig 5*A* shows the average DIC scores ±SE across all observers in each task relative to the ideal Bayesian model. A score above zero indicates a better fit. For additional analysis at the group level, we obtained the exceedance probability (*φ*_*k*_) in favor of each model *k* in the covert- (Table 1) and overt-criterion (Table 2) tasks. The exceedance probability for each model is shown in Fig 5*B*.

**Table 1:** Individual DIC scores for the covert-criterion task under static conditions (Expt. 1).

Observer	Ideal Bayesian	Bayesian model selection	Exponentially weighted moving-average	Reinforcement-learning	Limited memory
ADB	4,940	458	415*	423	471
DJA	5,020	580	551*	569	573
DMG	5,080	564	557*	559*	666
EHN	4,809	328	288*	331	436
EKC	4,964	492*	495*	496*	601
ID	5,043	630	594*	632	613
JYZ	4,930	403*	413	405*	496
LD	4,973	479	490	464*	581
MR	5,179	745	722*	744	723*
SJ	5,008	572	560*	574	593
Mean	4,995	525	509*	520	575
Exceedance probability (*φ*)	.003	.02	.95	.02	.003

Note: The * indicates the best fitting model (i.e., the model with the lowest DIC score) for each observer and the average across all observers. We allow for ties.

**Table 2:** Individual DIC scores for the overt-criterion task under static conditions (Expt. 1).

Observer	Ideal Bayesian	Bayesian model selection	Exponentially weighted moving-average	Reinforcement-learning	Limited memory
ADB	7,966	3,516	3,130	3,380	2,936*
DJA	8,237	3,778	3,326	3,299*	3,446
DMG	9,019	4,485	4,436*	4,454	4,601
EHN	7,942	3,418	3,167*	3,226	3,690
EKC	8,543	4,073	3,920*	3,923*	4,135
ID	7,935	3,451	3,240*	3,379	4,669
JYZ	9,083	4,610	4,576	4,551	3,356*
LD	8,403	3,988	3,404*	3,425	3,501
MR	8,533	4,174	3,951*	4,072	4,071
SJ	8,416	3,912	3,887	3,598*	4,153
Mean	8,408	3,941	3,704*	3,731	3,856
Exceedance probability (*φ*)	.004	.004	.78	.19	.02

Note: The * indicates the best fitting model (i.e., the model with the lowest DIC score) for each observer and the average across all observers. We allow for ties.

For 5 out of 10 observers in the covert-criterion task, the exponentially weighted moving-average model fit the best. Of the remaining five observers, one was fit equally well by the exponentially weighted moving-average and the limited-memory models, one was fit best by the Bayesian selection, exponentially weighted moving-average, and the reinforcement learning models, one was fit best by the Bayesian selection and the reinforcement learning models, one was fit best by the exponentially weighted moving-average and reinforcement learning models, and one was best fit by the reinforcement learning model. At the group level, the exceedance probability for the exponentially weighted moving-average is very high (*φ*_Exponential_ = .95) suggesting that given the group data, it is a more likely model than the alternatives (Table 1).

In the overt-criterion task, the exponentially weighted moving-average model fit best for 5 out of 10 observers. Of the remaining five observers, one was fit equally well by the exponentially weighted moving-average and the reinforcement learning models, two were fit best by the reinforcement-learning model, and two were fit best by the limited-memory model. At the group level, the exceedance probability for the exponentially weighted moving-average model (*φ*_Exponential_ = .78) is higher than the alternatives suggesting that it is more likely given the group data (Table 2).

**Fig 6:**
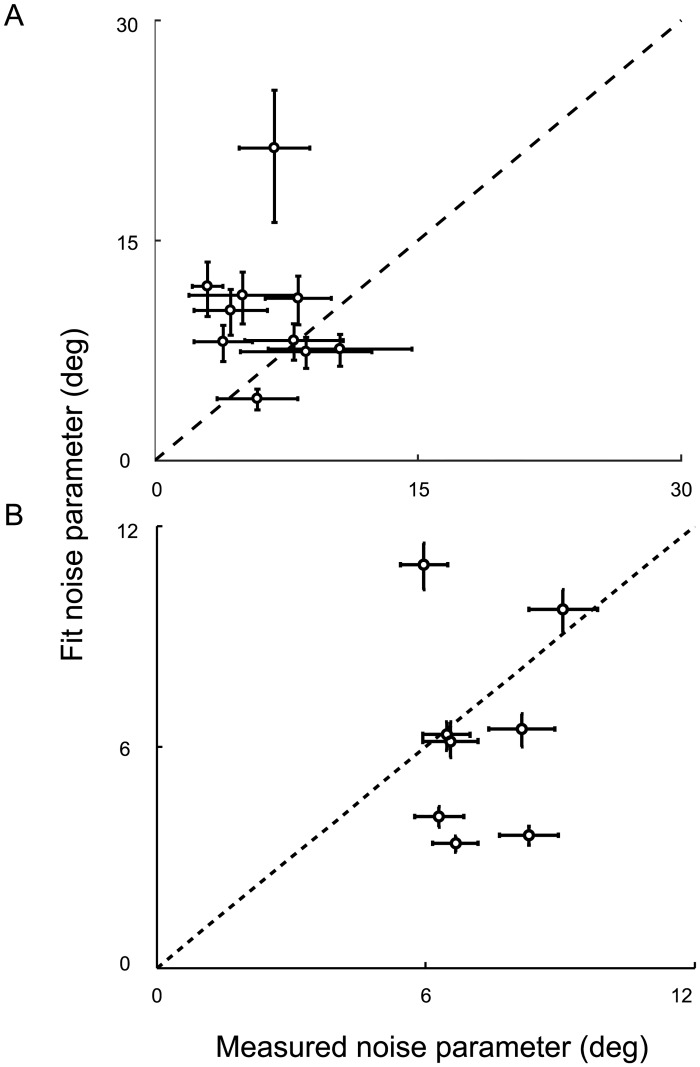
A comparison of the measured noise parameters and model fit parameters in Expt. 1 for the exponentially weighted moving-average model. **A**) Each model was fit to the covert-criterion data for each individual and the maximum a posteriori parameter estimate for sensory noise (*σ*_*v*_) was determined. Each point represents the sensory noise estimated by the exponentially weighted moving-average model for each individual compared to the individual’s measured sensory noise. Black dashed line: the identity line. **B**) Adjustment noise (*σ*_*a*_) was estimated in the overt-criterion task and compared to the measured adjustment noise. Note: adjustment noise was only measured for 8 out of the 10 observers. Error bars represent a 95% C.I.

Parameter estimates for each model and task are shown in Tables 3 and 4. Additionally, Fig 6 compares the noise-parameter estimates (*σ*_*v*_ or *σ*_*a*_) for the exponentially weighted moving-average model to the sensory and adjustment noise that were measured in the orientation-discrimination and orientation-matching tasks, respectively. Generally, fit parameters were larger than the corresponding estimates from the discrimination experiment and smaller than the corresponding estimates from the matching experiment. We computed correlations between the measured sensory noise estimates and each model’s estimates in the covert-criterion task and found no significant correlation between them for any of the models (all *p* > .05). No significant correlation was found when comparing measured adjustment noise and model estimates either (all *p* > .05). This suggests that the fitted noise parameters reflected an additional noise source in the covert-criterion task that dominated the sensory variability (e.g., uncertainty about the distribution noise). In the overt-criterion task, the fitted noise may have been smaller because the current criterion setting was highly correlated with previous settings, which was not true in the matching task.

**Table 3:** Mean maximum a posteriori parameter estimates ±SE for each covert-criterion model in Expt. 1.

Model	*σ*_*v*_	*σ*	*τ*	*β*
Ideal Bayesian	10.5 ± 1.4	10.0 ± .09	—	—
Bayesian model selection	10.5 ± 1.5	—	—	—
Exponentially weighted moving-average	10.1 ± 1.4	—	[2.2, 4.2]	—
Reinforcement learning	10.5 ± 1.5	—	—	.11 ± .04
Limited memory	13.6 ± 1.3	—	—	—

**Table 4:** Mean maximum a posteriori parameter estimates ±SE for each overt-criterion model in Expt. 1.

Model	*σ*_*a*_	*σ*	*τ*	*β*
Ideal Bayesian	6.9 ± .77	9.9 ± .14	—	—
Bayesian model selection	6.8 ± .76	—	—	—
Exponentially weighted moving-average	5.8 ± .86	—	[3.2, 4.5]	—
Reinforcement learning	5.3 ± .85	—	—	.41 ± .04
Limited memory	6.6 ± .97	—	—	—

### Experiment 2

In Expt. 2, observers completed the same tasks in Expt. 1. However, the environment was dynamic and only 3 out of 10 observers completed the orientation-matching task. Category distributions were Gaussian distributions with different mean orientations and equal variance, but means of the category distributions changed gradually over time via a random walk (see Methods).

#### Estimating sensory uncertainty

The same orientation-discrimination task that was used to estimate sensory uncertainty in Expt. 1 was also used in Expt. 2. On average, *σ*_*v*_ = 9.2° with individual observer thresholds ranging from 5.0 to 14.2°.

#### Raw overt-criterion data

**Fig 7:**
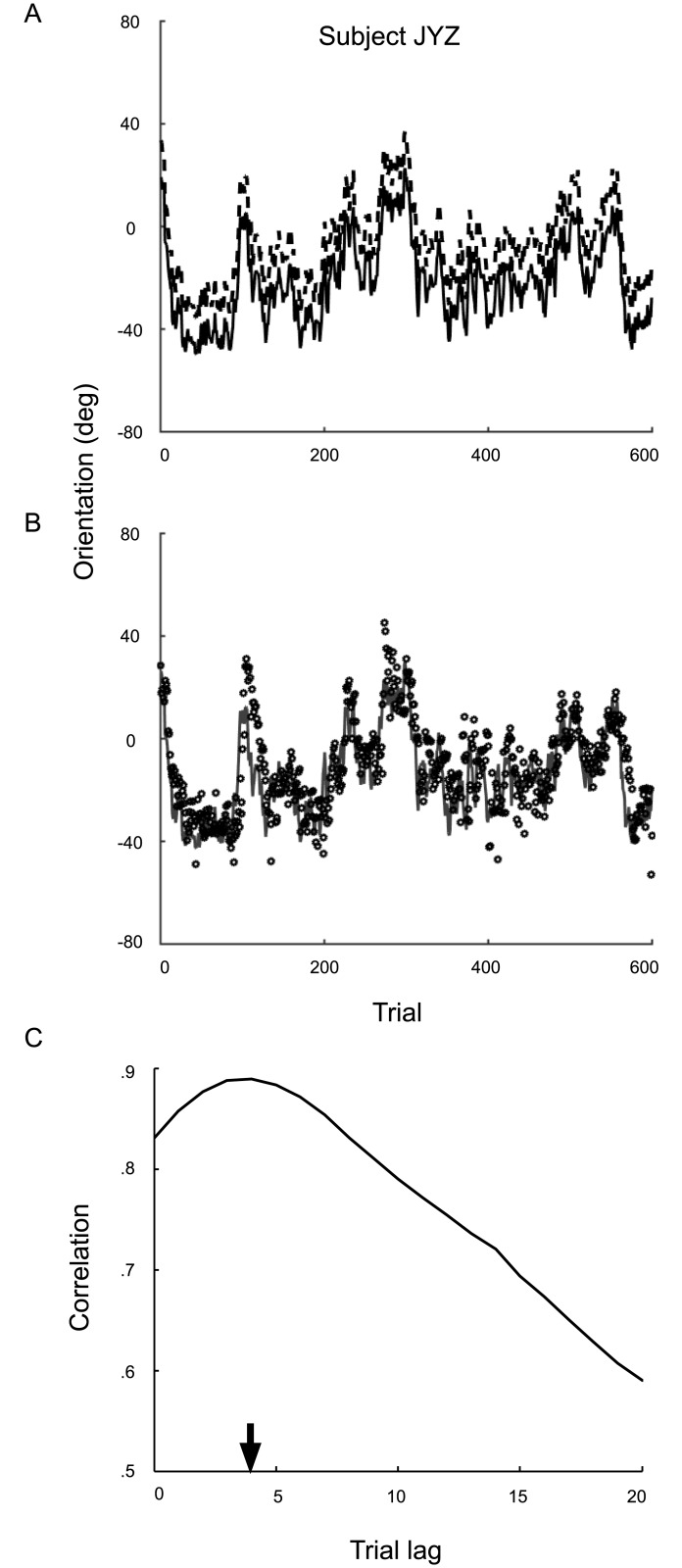
Overt-criterion data for two observers in Expt. 2. **A**) The mean positions of the category *A* (solid line) and *B* (dashed line) across the overt-criterion block. **B**) Criterion placement data across the block (data points) compared to the omniscient criterion placement (solid gray line). **C**) Cross-correlation between the omniscient criterion and the observer’s criterion placement. The lag estimate is indicated by the arrow. Estimated lags for all observers ranged from 1 to 4.

Results for a sample observer in Expt. 2 are shown in Fig 7. Fig 7*A* shows the random walk that the means of category *A* (solid black line) and category *B* (dashed black line) followed across the block. We estimated how well each observer tracked the changes in the mean by cross-correlating the observer’s criterion settings (data points in Fig 7*B*) with the omniscient criterion (gray line in Fig 7*B*, the average of the two actual category means). The peak of the correlation function (Fig 7*C*) provides an estimate of the lag in response to the changing category means. Peak lags ranged from 1 to 4 across observers, indicating that they were able to closely follow the changes in the mean.

#### Response to recently experienced stimuli

**Fig 8:**
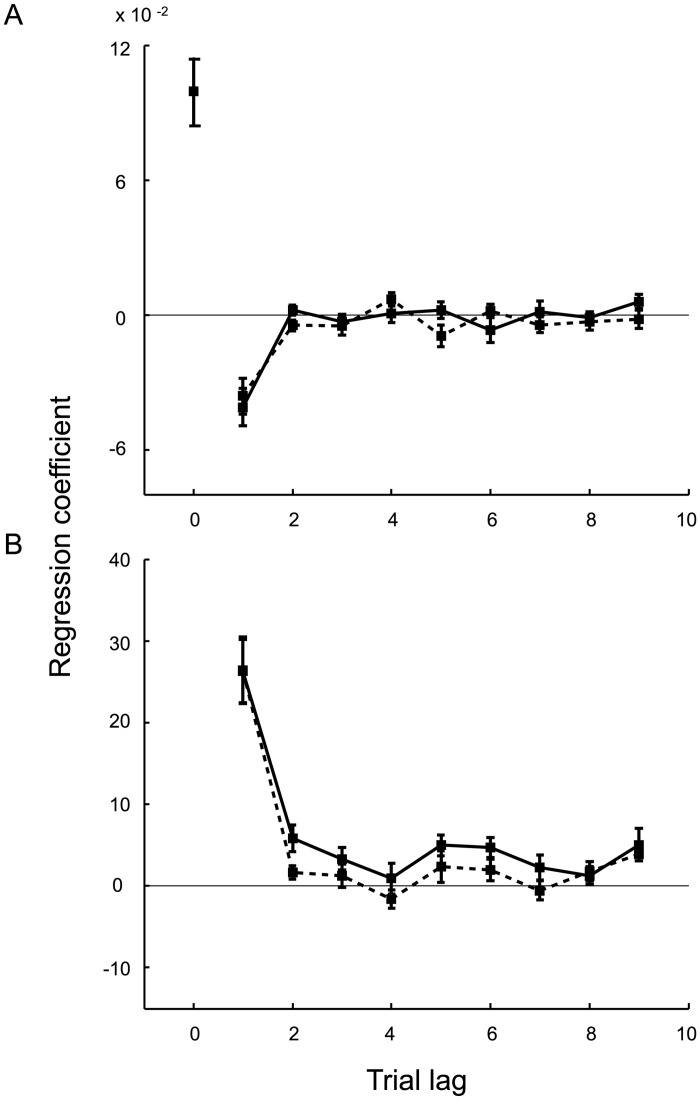
Lagged regression for the dynamic condition (Expt. 2). **A**) Covert-criterion task: Results of a logistic regression predicting the binary decision of each trial as a combination of the orientations of the current ellipse and the previous nine ellipses in each category. The solid and dashed lines represent the group average beta weights ±SE for the ellipses belonging to category *A* and category *B*, respectively. **B**) Overt-criterion task: Results of a linear regression predicting the criterion placement on each trial as a combination of the orientation of the previous nine ellipses in each category. Again, the solid and dashed lines represent the group average beta weights ±SE for the ellipses belonging to category *A* and category *B*, respectively.

As in Expt. 1, we conducted “lagged regressions” on the data from each task. We included the orientations of the nine most recently experienced ellipses from each category as regressors. As in the lagged regression results for Expt. 1, the overall trend appears to weight recent ellipses more heavily than those further in the past (Fig 8). We observed a sign change for the covert-criterion task (Fig 8*A*), where the weight on lag one was a fraction of the weight on lag zero ($\frac{\beta_{A,\;n-1}}{\beta_{A,\;n}}=-.41$ and $\frac{\beta_{B,\;n-1}}{\beta_{B,\;n}}=-.37$). This suggests that the current decision was determined by taking the difference between the current stimulus and a weighted average of the previous stimuli (i.e., the criterion). Regression weights for the overt-criterion task (Fig 8*B*) are exponential in shape, suggesting again that the current criterion setting is a weighted average of the previous stimuli. The higher value of the beta weights for the most recent stimuli in Expt. 2 compared to Expt. 1 suggests that the exponential weights have a shorter time constant, as would be predicted for, e.g., a Kalman filter given the more volatile time series for the category means (a random walk vs. a constant).

In the analysis of the covert-criterion data, the average influence of category *A* ellipses differed significantly from zero for two of the nine lags (*p* < 0.05) and the average influence of category *B* ellipses differed significantly from zero for four of the nine lags (*p* < 0.05). In the overt-criterion block, the average influence of category *A* ellipses differed significantly from zero for six of the nine lags (*p* < 0.05) and the average influence of category *B* ellipses differed significantly from zero for two of the nine lags (*p* < 0.05).

#### Model comparison

Due to the complexity of the ideal-observer model in a dynamic environment, the ideal-observer model was excluded from the model comparison. Additionally under dynamic conditions, the Bayesian selection model is indistinguishable from the exponentially weighted moving-average model (see Methods). Thus, the model comparison for Expt. 2 begins with the exponentially weighted-moving average model.

**Fig 9:**
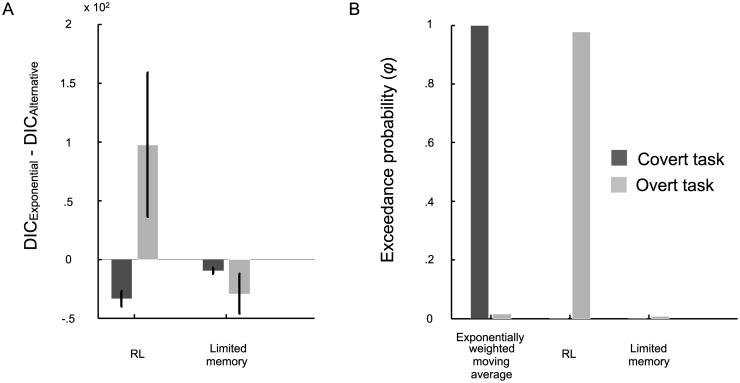
Model comparison results for the covert- (dark gray) and overt-criterion (light gray) tasks in Expt. 2. **A**) The bar graph depicts the relative DIC scores (i.e., DIC difference between the exponentially weight moving-average model and the alternatives) averaged across observers ±SE. Larger values indicate a better fit. **B**) To summarize the results from the group level analysis we computed exceedance probabilities for each model in each task. A model’s exceedance probability tells us how much more likely that model is compared to the alternatives, given the group data.

To obtain a quantitative measure of model fit, we computed DIC scores for each combination of task, observer and model. The median DIC scores for each observer and model in the covert- and the overt-criterion task are displayed in Tables 5 and 6, respectively. The best fitting model for each observer is indicated. We allowed for ties, so occasionally multiple models were the best fitting and are indicated as such. Fig 9*A* shows the average DIC scores across all observers in each task relative to the DIC scores for the exponentially weighted average model. A score above zero indicates a better fit. For additional analysis at the group level, we obtained the exceedance probability (*φ*_*k*_) in favor of each model *k* in the covert- (Table 5) and overt-criterion (Table 6) tasks. The exceedance probability for each model is shown in Fig 9*B*.

**Table 5:** Individual DIC scores for the covert-criterion task under dynamic conditions (Expt. 2).

Observer	Exponentially weighted moving-average	Reinforcement learning	Limited memory
ADB	602*	660	622
ASD	747*	792	746*
BAC	629*	639	632*
DJA	789*	840	791*
DMG	702*	724	714
EHN	486*	545	487*
ERK	713*	719*	725
JMP	701*	707*	723
JYZ	643*	688	657
MLN	565*	600	581
Mean	658*	691	668
Exceedance probability (*φ*)	.9985	.0006	.001

Note: The * indicates the best fitting model (i.e., the model with the lowest DIC score) for each observer and the average across all observers. We allow for ties.

**Table 6:** Individual DIC scores for the overt-criterion task under dynamic conditions (Expt. 2).

Observer	Exponentially weighted moving-average	Reinforcement learning	Limited memory
ADB	3,975*	4,014	3,973*
ASD	4,935	4,725*	4,898
BAC	4,590	4,462*	4,645
DJA	4,558	4,284*	4,550
DMG	4,517	4,263*	4,620
EHN	4,206	3,907*	4,217
ERK	4,033	3,959*	4,081
JMP	3,877*	4,229	3,906
JYZ	3,911*	3,905*	4,035
MLN	4,397	4,278*	4,368
Mean	4,300	4,203*	4,329
Exceedance probability (*φ*)	.015	.978	.007

Note: The * indicates the best fitting model (i.e., the model with the lowest DIC score) for each observer and the average across all observers. We allow for ties.

The covert-criterion data for 4 out of 10 observers were best fit by the exponentially weighted moving-average model. Four observers were best fit by the exponentially weighted moving-average and the limited-memory models and two were best fit by the exponentially weighted moving-average and reinforcement learning models. At the group level, the exceedance probability for the exponentially weighted moving-average model was much higher (*φ*_Exponential_ = .9985) than either alternative model suggesting it is a more likely model given the group data (Table 5).

In the overt-criterion task, 7 out of 10 observers were best fit by the reinforcement learning model. Of the remaining three, one was best fit by the exponentially weighted moving-average model, one was fit equally well by the exponentially weighted moving-average and reinforcement learning models, and one was fit equally well by the exponentially weighted moving average and the limited-memory models. At the group level, the exceedance probability in favor of the reinforcement learning model is very high (*φ*_*RL*_ = .978) suggesting that given the group data, the reinforcement learning model is more likely compared to either alternative (Table 6).

**Fig 10:**
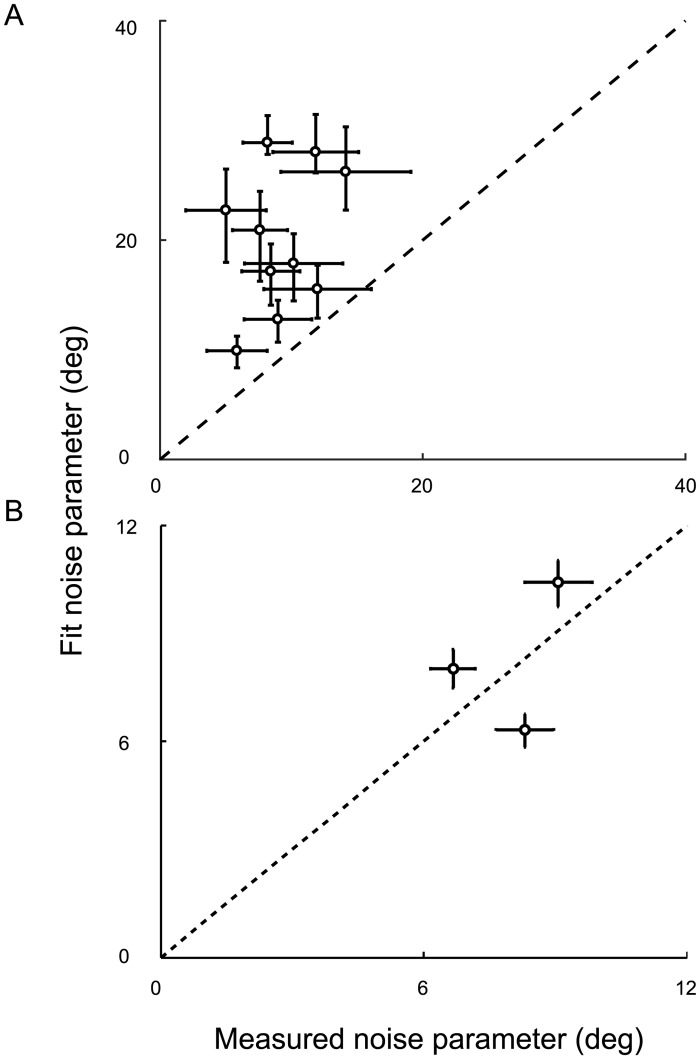
A comparison of the measured noise parameters and model fit parameters in Expt. 2 for the exponentially weighted moving-average model. **A**) Each model was fit to the covert-criterion data for each individual and the maximum a posteriori parameter estimate for sensory noise (*σ*_*v*_) was determined. Each point represents the sensory noise estimated by the exponentially weighted moving-average model for each individual compared to the individual’s measured sensory noise. Black dashed line: the identity line. **B**) Adjustment noise (*σ*_*a*_) was estimated in the overt-criterion task and compared to the measured adjustment noise. Error bars represent a 95% C.I. Note: adjustment noise was only measured for 3 out of the 10 observers, who completed both Expt. 1 and Expt. 2.

Parameter estimates for each model and task are shown in Tables 7 and 8. Additionally, Fig 10 compares the noise-parameter estimates for the exponentially weighted moving-average model to the sensory and adjustment noise that were measured in the orientation-discrimination and orientation-matching tasks, respectively. Adjustment noise was only measured for 3 of the 5 observers who completed both experiments. For the covert-criterion task, fit parameters are larger than the corresponding noise estimates from the discrimination experiment. For the overt-criterion task, the fit parameters are larger than the corresponding noise estimates from the matching experiment for 2 out of 3 observers and smaller for one. Larger parameter estimates might indicate additional uncertainty in the task (e.g., uncertainty about the distribution noise and/or uncertainty about the dynamics of the task). Correlations between the measured sensory noise estimates and the estimates from each model fit revealed no significant correlation between the two (all *p* > .05). No significant correlation was found when comparing measured adjustment noise and model estimates either (all *p* > .05).

**Table 7:** Mean maximum a posteriori parameter estimates ±SE for each covert-criterion model in Expt. 2.

Model	*σ*_*v*_	*τ*	*β*
Exponentially weighted moving-average	20.0 ± 2.1	[.81, 1.06]	—
Reinforcement learning	23.2 ± 1.8	—	.56 ± .07
Limited memory	21.3 ± 2.0	—	—

**Table 8:** Mean maximum a posteriori parameter estimates ±SE for each overt-criterion model in Expt. 2.

Model	*σ*_*a*_	*τ*	*β*
Exponentially weighted moving-average	9.1 ± .93	[1.1, 1.6]	—
Reinforcement learning	8.3 ± .65	—	.79 ± .06
Limited memory	9.3 ± .89	—	—

## Discussion

The present study examined the strategies observers used to learn and update their decision criterion in an orientation-discrimination task under both static and dynamic uncertainty. Under static conditions in which category means were constant, we showed that while observers converged to the optimal criterion over many trials, their trial-by-trial behavior was better described by suboptimal learning rules than by the optimal rule. Thus, even though conditions were static, the criterion continued to systematically drift with changes in stimulus statistics throughout the experiment. Under dynamic conditions in which category means changed slowly over time, observers followed changes in the means of the category distributions closely with a 1–4 trial lag. Specifically, we found that at the group level a model in which the recent history of past samples determines a belief about category means, the exponentially weighted moving-average rule, was more likely than the alternative models across most tasks and conditions with the exception of the overt-criterion task under dynamic conditions in which the reinforcement learning model was more likely. Our results suggest that the decision criterion is not fixed, but is dynamic, even after prolonged training. Finally, we provided a novel technique, the overt-criterion task, which can be used to explicitly measure criterion placement and a computational framework for decision-making under uncertainty in both static and dynamic environments.

Based on findings in the visuo-motor and reinforcement-learning literature, in which feedback is gradually or discretely updated [15,16,17,20,21], we would expect a model in which recent samples are given more weight to better explain performance under dynamic conditions. However, this is a suboptimal strategy under static conditions. Nevertheless, research on history effects in psychophysical tasks suggests that observers behave as if the environment is dynamic, which is consistent with our results [8,9,10,11,12,13,14]. Furthermore, the regression analysis we performed in Expt. 1 revealed beta weights for the covert- and overt-criterion tasks that suggest an exponentially weighted average of the previous stimuli. Overall, our analysis provides additional evidence against the ideal-observer model and the assumption of a stable criterion, even in static environments. Intuitively, in a world that is constantly changing, it makes sense to continually update your decision criterion, weighting your most recent experiences more heavily.

The previous study that is closest in spirit to the current work is that of Summerfield and colleagues [19]. Their experiment was similar to ours; in their case category means changed suddenly at every 10 or 20 trials, and category variances could also change. However, they used a traditional discrimination task without explicit measurement of the criterion, and their primary analysis used the predictions of each of three models in a decidedly suboptimal manner: probability matching. They found two extremely different models, a limited-memory model and a Bayesian model (that uses probability matching rather than the optimal decision) both accounted for significant amounts of variance in their data. In our analysis, we are interested in the entire sequence of computations from estimating the stimulus parameter of interest (orientation, perturbed by sensory noise) through the binary category decision, and compare a wider array of models that include the ideal observer. We found that suboptimal models that use the recent history of past samples best accounted for both covert and explicit criterion setting [19].

While a dynamic decision criterion might be useful in the real-world, by using such a criterion (especially in Expt. 1) in our experiments, observers are making suboptimal inferences about the category membership of an ellipse. These results are consistent with the idea that suboptimal inference is more than just internal noise [22]. This is also consistent with the overestimates of the noise parameters that we find in our model fits, which suggests that there is additional noise beyond sensory and adjustment. Acuña and Schrater [23] suggest that seemingly suboptimal decisions in sequential decision-making tasks can be accounted for by uncertainty in learning the structure of the task. Uncertainty about the structure of the environment could affect observers’ criterion placement (i.e., observers might be uncertain as to whether the category parameters are changing and/or the rate of change). In novel situations, one must learn the task structure and the parameters of the environment to perform optimally.

For the purpose of increasing statistical power for our model comparison, we introduced a novel task, in which we made the decision criterion explicit. Previous research suggests that participants change strategies when implicit tasks are made explicit [24,25,26]. Specifically, participants who perform optimally during an implicit task are not optimal when the task is made explicit. This is thought to be a result of higher-level strategic adjustments interfering with lower-level processing. While strategies were fairly consistent under static conditions, we found a clear difference in preferred strategy under dynamic conditions. Specifically, we found the exponentially weighted moving-average model fit best in the covert-criterion task and the reinforcement learning model fit best in the overt-criterion task. Additionally, we observed a difference in the exponentially weighted moving-average model’s decay rate and the reinforcement learning model’s learning rate. Across experiments, the decay and learning rates under static conditions were slower than decay and learning rates under dynamic conditions. However, there was also a difference across tasks. In both experiments, the decay rate was slower in the overt- than the covert-criterion task and the learning rate was faster in the overt- than covert-criterion task. Since a slower decay rate is beneficial under static conditions but disadvantageous under dynamic conditions and a faster learning rate is beneficial under dynamic conditions but disadvantageous under static conditions, the parameter differences we observed might explain the differences we see in the preferred strategies used across tasks. In particular, this may explain why the reinforcement learning model performed better than the exponentially weight moving-average model in the overt-criterion task under dynamic conditions. The differences in decay and learning rate between the covert- and overt-criterion tasks might be due to a difference in time-scale that results from the temporal dynamics of the two tasks (the overt-criterion task took twice as long to complete the same number of trials) or due to the different levels of processing (e.g., sensory vs. motor) required for each task. In the future, it might be interesting to see how the decay and learning rates trade off as a function of the rate of change (i.e., the random-walk variance) in the experiment.

Previous research shows that participants update the decision criterion when changes to the prior probabilities and payoff matrix occur [1,2,3,4,5]. There is a systematic bias in these shifts: Humans exhibit conservatism, that is, a bias towards the neutral criterion when the optimal criterion is shifted away from neutral. While several hypotheses have been proposed as to why conservatism occurs, most recently Ackermann and Landy [2] have suggested that conservatism can be explained by distorted probability and utility functions. Our results do not explain this bias, but it is likely that conservatism is present and contributes to the dynamics of trial-by-trial criterion shifts under the conditions of Expt. 2. To provide a better understanding of this bias, further research should aim to examine criterion learning in situations in which conservatism is known to exist.

Finally, psychophysical studies rely heavily on accurate estimates of *d*′. By calculating *d*′ from hit rates and false alarms in the usual way, a fixed criterion is assumed. However, if the observer’s criterion varies over trials, performance will be a mixture of multiple points on the ROC curve, resulting in a biased (too-low) estimate of *d*′. We have shown here that decision criteria are adjusted dynamically. Examining the dynamics of trial-by-trial criterion placement provides us with a richer understanding of participants’ behavior when making decisions in the presence of uncertainty. Our results suggest that typical estimates of *d*′ are biased, and that by using a model that accounts for a dynamic criterion we can compute a more accurate measure of discriminability and in turn, obtain a more comprehensive understanding of discrimination under uncertainty.

## Methods

### Ethics statement

This research involved the participation of human subjects. The Institutional Review Board at New York University approved the experimental procedure and observers gave informed consent prior to participation.

### Participants

Ten observers participated in Expt. 1 (mean age 25.4, range 20–33, 5 females) and Expt. 2 (mean age 23.4, range 20–28, 4 females). Five observers provided data for both experiments, three of whom completed Expt. 1 prior to completing Expt. 2. All observers had normal or corrected-to-normal vision. One of the observers (EHN) was also an author.

### Apparatus

Stimuli were presented on a gamma-corrected Dell Trinitron P780 CRT monitor with a 31.3 x 23.8 deg display, a resolution of 1024 x 768 pixels, a refresh rate of 85 Hz, and a mean luminance of 40 cd/m^2^. Observers viewed the display from a distance of 54.6 cm. The experiment was programmed in MATLAB (MathWorks) using Psychophysics Toolbox [27,28].

### Stimuli

Stimuli were 10 x 2° ellipses presented at the center of the display on a mid-gray background (Fig 1). In the orientation-matching and overt-criterion tasks, a yellow line was presented at the center of the display (10 x .35°). In all tasks except the overt-criterion task, trials began with a central yellow fixation cross (1.2°).

### Experiment 1

Ten observers participated in one, 1.5-hour session consisting of an orientation-discrimination task (~10 min), a covert- and an overt-criterion practice block (~5 min combined), one block of the covert-criterion task (~20 min), and one block of the overt-criterion task (~40 minutes). The order of the covert- and overt-criterion tasks was randomized across subjects. Eight out of ten observers returned for a second session in which they completed an orientation-matching task (~20 minutes). Before starting the experiment observers were given detailed instructions regarding the tasks they would be asked to complete. The two short (20 trial) practice blocks were used to ensure that observers understood the experimental tasks. Before each block, a condensed version of the instructions and the name of the task were shown to remind observers of the procedure and inform them of the task they would be completing on that block.

#### Orientation-discrimination task

To estimate sensory uncertainty (i.e., the just noticeable difference in ellipse orientation), observers performed a two-interval forced-choice procedure in which two black ellipses were presented sequentially (Fig 1*A*). The observer’s task was to report the interval containing the ellipse that was more clockwise by pressing 1 or 2 on the keypad, respectively. After the response, auditory feedback was provided and the next trial began.

The orientation of the ellipse in the first interval was chosen randomly on every trial from a uniform distribution (*θ*_1_~*unif*(−74°,74°)). The orientation of the second ellipse (*θ*_2_) was randomly oriented clockwise or counter-clockwise of the first by an amount Δ*θ* ranging from .25°-16° (log-spaced). The difference in orientation between the two ellipses was selected using an adaptive staircase procedure. Four staircases (65 trials each) were interleaved (two 1-up, 2-down and two 1-up, 3-down) and randomly selected on each trial [29].

#### Orientation-matching task

To estimate orientation-adjustment uncertainty, participants performed an orientation-matching task (Fig 1*B*). A yellow line was briefly presented in the center of the display. The observer’s task was to rotate a subsequently presented line (initially vertical) by dragging it with the mouse to match the orientation of the first line. When satisfied with the orientation setting, observers clicked on an enter button (not visible during adjustments of the line) at the bottom of the screen and the next trial began. Line orientation was chosen randomly and uniformly (*θ*_1_~*unif*(−90°,90°)). The block consisted of 260 trials.

#### Covert-criterion task

In the covert-criterion task (Fig 1*C*), observers were shown a black ellipse and indicated to which of two categories (*A* or *B*) it belonged by key press. Observers were told that there were two categories of ellipse, green and red, and that the mean of the green category (*μ*_*A*_) was always clockwise of the mean of the red category (*μ*_*B*_). The ellipse was equally likely to belong to either category. Auditory feedback indicated whether the response was correct and visual feedback indicated the ellipse’s true category membership: The fixation cross was shown in the color of the correct category. The observer received a point for each correct response. The total score was shown at the top of the display along with the feedback. After the feedback, the next trial began automatically. The block consisted of 600 trials.

Ellipse orientation was chosen randomly from one of two overlapping Gaussian distributions representing the two categories of ellipses (*θ*_*A*_~*N*(*μ*_*A*_,*σ*) and *θ*_*B*_~*N*(*μ*_*B*_,*σ*)), where *σ* = 10° and *μ*_*A*_ < *μ*_*B*_ (Fig 1E). At the beginning of the block, *μ*_*A*_ was randomly selected from a uniform distribution ranging from approximately -50° to 50°. Finally, to equate the difficulty of the task across observers, *μ*_*B*_ was rotated counter-clockwise from *μ*_*A*_ by an amount Δ*θ* that corresponded to *d*′ = 1 as estimated using the data from the orientation-discrimination task. The overlap of these distributions introduced ambiguity: A given orientation could come from either category and therefore categorization performance could not be perfect even in the absence of sensory noise.

#### Overt-criterion task

In the overt-criterion task (Fig 1*D*), observers were required to indicate their decision criterion explicitly on each trial. On each trial, observers adjusted the orientation of a “criterion line” by dragging it with the mouse to best separate the green and red ellipse categories. As in the covert-criterion task, they were informed that there were two noisy categories of ellipses and that, on average, the green ellipses were clockwise of the red. On each trial, the ellipse was equally likely to belong to either category. When satisfied with the setting, observers clicked the enter button at the bottom the display. Then, either a green or red ellipse appeared under the line and auditory feedback was provided. If the ellipse was green and clockwise of the line or red and counter-clockwise of the line the observer received positive auditory feedback and one point. Otherwise, the observer received negative auditory feedback and no points. The total score was shown at the top of the display along with the feedback. Category *A* (green) and *B* (red) means and variances were chosen in the same manner but the means were independent of those chosen for the covert-criterion block. Observers had to relearn the categories at the beginning of each block. The block consisted of 600 trials.

### Experiment 2

Expt. 2 was similar to Expt. 1 except that observers did not complete the orientation-matching task and the category distribution means in the covert- and overt-criterion tasks were not constant throughout the block. Rather, category means were updated on every trial following a random walk. The category *A* mean on trial *n*+1 was *μ*_*A*,*n+1*_ = *μ*_*A*,*n*_+*ε*, where *ε* ~*N*(0,*σ*_random_) and *σ*_random_ = 5°. The relative position (*μ*_*A*_ < *μ*_*B*_) and the distance between the means remained constant.

### Computational models

In the covert-criterion task, the statistical structure of the task involves three variables: category *C*, stimulus orientation *S*, and measurement *X*. On each trial, *C* is drawn randomly and determines whether *S* is drawn from category *A* (*N*(*μ*_*A*,_*σ*)) or category *B* (*N*(*μ*_*B*,_*σ*)). We assume that on each trial, the true orientation is corrupted by sensory noise (with standard deviation *σ*_*v*_) to give rise to the observer’s measurement of orientation (*X*~*N*(*S*,*σ*_*v*_)). The observer uses this measurement to infer the category.

In the overt-criterion task, the statistical structure of the task involves five variables: criterion orientation *θ*_*c*_, criterion placement *z*, category *C*, stimulus orientation *S*, and measurement *X*. On each trial, criterion orientation is inferred from the previous trials. We assume that criterion orientation is corrupted by adjustment noise (*z*~*N*(*θ*_*c*_,*σ*_*a*_)). After the criterion is set, *C* is drawn randomly and determines whether *S* is drawn from category *A* (*N*(*μ*_*A*,_*σ*)) or category *B* (*N*(*μ*_*B*,_*σ*)). As in the covert-criterion task, we assume the true orientation of the stimulus is corrupted by sensory noise (*X*~*N*(*S*,*σ*_*v*_)). Finally, the observer uses this measurement and the feedback about its category membership to update the criterion orientation for the next trial. We found that model fits for the overt case could not discriminate adjustment noise (*σ*_*a*_) from sensory noise (*σ*_*v*_), and so for this case, sensory noise was fixed and only an adjustment noise parameter was fit. Sensory noise was set to each observer’s measured sensory uncertainty. Below we describe both optimal and suboptimal models of criterion learning that vary in computational and memory demands. The selection of the following models was partially inspired by the models used in Summerfield and colleagues’ research [19] investigating perceptual classification strategies in rapidly changing environments, in which they compared a Bayesian observer model to a Q-learning model and a heuristic model that is similar to our limited-memory model. In their models, sensory noise is omitted, and in its place, a fixed degree of trial-trial choice variability is introduced by a probability-matching rule. In contrast, we compare a more extensive set of models that include parameters controlling the level of sensory noise and predict a specific response based on the noisy stimulus measurement and a model of criterion update.

#### Ideal observer

For the covert-criterion task, the ideal Bayesian observer decides to which category the current sample *X*_*n+1*_ belongs by computing the posterior probability that the observation belongs to each category given the noisy measurements of all previously observed samples, integrating across the unknown category parameters. For the static case (Expt. 1) the posterior odds ratio is a sufficient statistic:

$$\frac{P(A|X_{1},\ldots ,X_{n+1})}{P(B|X_{1},\ldots ,X_{n+1})}\propto \frac{p_{A}\int P(X_{n+1}\left|\Theta_{A})P(X_{A,1:n_{A}}\right|\Theta_{A})P(X_{B,1:n_{B}}|\Theta_{B})p(\Theta_{A},\Theta_{B})d\Theta }{(1-p_{A})\int P(X_{n+1}\left|\Theta_{B})P(X_{A,1:n_{A}}\right|\Theta_{A})P(X_{B,1:n_{B}}|\Theta_{B})p(\Theta_{A},\Theta_{B})d\Theta },$$ (1)

where $\Theta =(\Theta_{A},\Theta_{B})=(\mu_{A},\sigma_{A}^{2},\mu_{B},\sigma_{B}^{2})$, ${\text{X}}_{A,1:n_{A}}$ and ${\text{X}}_{B,1:n_{B}}$ are all of the previously observed noisy samples from category *A* and category *B* (*n* = *n*_*A*_ + *n*_*B*_), and *p*_*A*_ = 0.5. The integral takes into account the observer’s knowledge of the experimental conditions. In our case, we restricted the integral to parameter sets for which $\sigma_{A}^{2}=\sigma_{B}^{2}=\sigma^{2}$ and *μ*_*A*_ was clockwise of *μ*_*B*_. That is, the ideal observer was not privy to the knowledge of the exact amount by which *μ*_*A*_ differed from *μ*_*B*_. The prior on Θ was flat for the means over pairs for which *μ*_*A*_ was clockwise of *μ*_*B*_, and was a Jeffreys prior for the standard deviation (i.e., *P*(*σ*) ∝ 1/*σ*). The observer chooses category *A* when the ratio in Eq 1 is greater than 1.

In the overt-criterion task, the ideal observer chooses the criterion that maximizes the probability of being correct given all previously observed samples:

$$z_{n+1}=\underset{z}{\text{arg max}}P(\text{correct}|z,{\text{X}}_{1:n}).$$ (2)

This is equivalent to solving for the value of *X*_*n*+1_ for which the posterior odds ratio (Eq 1) is equal to one.

The ideal observer for the dynamic case (Expt. 2) is analogous to Eq 1. It marginalizes not only across a single set of mean and variance parameters, but rather across all trajectories the means might have followed in preceding trials. The complexity of this calculation is daunting, both for the human observer and even as a computer simulation. We don’t consider the ideal observer for the dynamic case below.

#### Bayesian model selection

An alternative suboptimal Bayesian approach is model selection, in which the most probable parameters are selected, and then decisions are made based on those values. For the static case, assuming flat priors and equal variance, the observer sets a criterion for the next trial by first computing the most likely estimates of the category means:

$$\begin{array}{ll} {\hat{\mu }}_{A,\;n+1} & =\frac{1}{n_{A}}\sum_{i=1}^{n_{A}}X_{A,\;i}\\ {\hat{\mu }}_{B,\;n+1} & =\frac{1}{n_{B}}\sum_{i=1}^{n_{B}}X_{B,\;i}. \end{array}$$ (3)

For the covert-criterion task, the observer chooses the category whose estimated mean is closest to the current observation. For the overt-criterion task, the observer places the criterion halfway between the estimates:

$$z_{n+1}=\frac{{\hat{\mu }}_{A,\;n+1}+{\hat{\mu }}_{B,\;n+1}}{2}.$$ (4)

By maintaining a running average, the observer does not have to keep track of every individual stimulus presentation but simply the last computed average and the total number of trials. On each trial, the new estimate of the mean is a weighted sum of the previous estimate and the current stimulus value:

$${\hat{\mu }}_{A,\;n+1}=\left(\frac{n_{A}}{n_{A}+1}\right){\hat{\mu }}_{A,\;n}+\left(\frac{1}{n_{A}+1}\right)X_{n+1},$$ (5)

and similarly for a current observation from category *B*.

For the dynamic case, model selection requires the observer to track individual category means. The Kalman filter [30] is the Bayesian mean-tracking rule for the random walk used in the dynamic experiment, and has been used to model human tracking of reward value [21] and consistent motor error [15]. Here, we briefly consider a Kalman-filter model in which the observer assumes a fixed variance for the random walk, which we fit to the data, rather than estimating it dynamically. In this model, suppose the observer assumes that category variance $\sigma_{A}^{2}=\sigma_{B}^{2}=\sigma^{2}$. Note that category variance cannot be discriminated from sensory variance, because both sources of noise are added to the underlying category means. If a category *A* ellipse is shown on trial *n*, the estimate of the category *A* mean is updated as follows,

$${\hat{\mu }}_{A,\;n+1}={\hat{\mu }}_{A,\;n}+\kappa_{n}\delta_{n},$$ (6)

where $\delta_{n}=X_{A,\;n}-{\hat{\mu }}_{A,\;n}$ is the prediction error, $\kappa_{n}=\sigma_{\text{total},\;n}^{2}/(\sigma_{\text{total},\;n}^{2}+\sigma_{A}^{2})$ is a trial-specific learning rate (or Kalman gain), $\sigma_{\text{total},\;n}^{2}=(1-\kappa_{n-1})\sigma_{\text{total},\;n-1}^{2}+\sigma_{\text{random}}^{2}$ and $\sigma_{\text{random}}^{2}$ is the observer’s estimate of the random-walk parameter. To start the process we set ${\hat{\mu }}_{A,\;1}=X_{A,\;1}$ and $\sigma_{\text{total},\;1}^{2}=0$. The sequence of Kalman gains *κ*_*n*_ depends only on the ratio $\sigma_{\text{random}}^{2}/\sigma_{A}^{2}$ and so we may fix $\sigma_{A}^{2}$ to the true value of the category variance, leaving $\sigma_{\text{random}}^{2}$ as a free parameter. A similar process estimates the category *B* mean. This is an error-driven learning model similar to temporal-difference learning or other delta-rule methods. The main difference is the additional tracking of uncertainty, which determines the trial-specific learning rate. After computing estimates of the category means, the criterion is set between the estimates (Eq 4). However, for this simple one-dimensional Kalman filter, for reasonable values of $\sigma_{\text{random}}^{2}/\sigma_{A}^{2}$ (e.g., ranging from 0.1 to 10), the Kalman gain rapidly asymptotes to a fixed value (within 5–20 trials). Thus, this model cannot be discriminated from a model with fixed gain, which is identical to the exponentially weighted moving-average model discussed next. Therefore, for the dynamic case, we begin with the exponential model.

#### Exponentially weighted moving-average

The exponentially weighted moving-average model computes smoothed estimates of the distribution means by taking a weighted average of the previously experienced stimuli for each category, where more recent stimuli are given more weight. The following geometric progression was used as a discrete form of an exponential weighting function:

$$\begin{array}{ll} {\hat{\mu }}_{A,\;0} & ∼unif(-90,\;90),\\ {\hat{\mu }}_{A,\;n+1} & =\alpha X_{A,\;n+1}+(1-\alpha ){\hat{\mu }}_{A,\;n}\;n>0, \end{array}$$ (7)

where *α* determines the exponential time constant and 0 < *α* < 1. A similar equation determines the estimate of the category *B* mean. When Eq 7 is expanded, the weights on the previous trials are proportional to the terms of the geometric progression 1,1−*α*,(1−*α*)^2^,⋯,(1−*α*)^*n−1*^. As *α* approaches 0, this model converges to the model-selection approach above, and as *α* approaches 1 this model converges to the limited-memory model below. On each trial, the observer places the criterion between the two estimates of the category means (Eq 4) for the overt-criterion task, and reports the category with the estimated mean closest to the current observation for the covert-criterion task. While theoretically different than the reinforcement-learning model described below (this model assumes the observer learns something about the category means), mathematically this model is equivalent to a reinforcement-learning model in which on each trial an estimate of a category mean is updated by a proportion of the difference between the current stimulus and the previous estimate of that category mean, such that for a category *A* trial, ${\hat{\mu }}_{A,\;n+1}={\hat{\mu }}_{A,\;n}+\alpha (X_{A,\;n}-{\hat{\mu }}_{A,\;n})$. Subsequently, the criterion is updated such that $z_{n+1}=z_{n}+\frac{1}{2}\alpha (X_{A,\;n}-{\hat{\mu }}_{A,\;n})$. The same holds for a category *B* trial. Importantly, category means are only updated when new information from the category is observed, but the criterion is updated on every trial.

As in the Bayesian model-selection strategy, the observer needs to remember the previous estimates of the category means. However, unlike the Bayesian model-selection strategy the observer does not need to remember the total number of trials observed in each category to combine the information. This model has two free parameters (*α* and *σ*_*v*_ or *σ*_*a*_). The time constant *τ* for the exponentially moving-average is a function of *α* and the sampling time interval Δ*T*, such that $\tau =\frac{-\Delta T}{\text{ln}(1-\alpha )}$. In our results, we report the time constant where Δ*T* = 1. To compute the time constant, *α* is estimated for each observer and averaged across individuals for a given condition. The average time constant is then computed using the average *α* parameter. The confidence interval on *τ* was determined by converting *α*±SE to *τ*.

#### Reinforcement learning (delta rule)

The reinforcement-learning model is a model-free approach that assumes the observer learns nothing about the underlying parameters of the distributions but simply interacts with the environment based on feedback. For both tasks, the observer updates an internal criterion (*z*_*n*_) on each trial according to the delta rule:

$$z_{n+1}=\{\begin{array}{ll} z_{n}, & \text{if correct}\\ z_{n}+\beta (X_{n}-z_{n}), & \text{if incorrect.} \end{array}$$ (8)

Thus, the criterion is updated when negative feedback is received by taking a small step in the direction of the difference between the stimulus sample and current criterion, where the step size is scaled by the learning rate *β*. For the overt-criterion task, the observer simply reports the current criterion. For the covert-criterion task, the current criterion is applied to the noisy observation. *β* is a free parameter that is fit for each observer.

#### Limited memory

In the limited-memory model, the observer only stores the most recently viewed samples of each category. The last sample drawn from each category is treated as the current estimate of the category mean:

$$\begin{array}{c} {\hat{\mu }}_{A,\;n+1}=X_{A,\;n_{A}}\\ {\hat{\mu }}_{B,\;n+1}=X_{B,\;n_{B}}. \end{array}$$ (9)

The criterion (*z*_*n+1*_) is placed halfway between the current estimates (Eq 4) and is either reported (overt task) or used to judge the next observation (covert task).

### Data analysis

#### Estimating sensory uncertainty from orientation-discrimination data

A cumulative normal distribution was fit to the orientation-discrimination data (probability of choosing interval one as a function of the orientation difference between the first and second ellipse) using a maximum-likelihood criterion [31]. From the fit curve we estimated the underlying sensory uncertainty (*σ*_*v*_) for each observer, which was compared to the estimates of sensory uncertainty from the fits of the computational models.

#### Estimating adjustment uncertainty from orientation-matching data

For each of the eight observers who completed the orientation-matching task, we calculated the adjustment error as the standard deviation (*σ*_*a*_) of the difference between the orientations of the observer’s setting and the previously displayed line. This value was compared to the estimates of adjustment variability from the fits of the computational models.

#### Regression analysis

In addition to the model comparisons, we also performed a model-free analysis of the data to determine the influence of prior trials on the current trial’s decision or criterion setting. We computed “lagged regressions” in which the regressors were the orientations of the nine most recently experienced ellipses from each category (that might have been used to estimate the category means). For the covert-criterion task, the current to-be-categorized ellipse’s orientation was also a regressor. The orientation of the current trial’s ellipse was not included for the overt-criterion task because the ellipse was always presented after the criterion was set. The dependent variable was either the decision (for the covert-criterion task) or the criterion placement (for the overt-criterion task). Because the dependent variable in the covert-criterion task was binary and the dependent variable in the overt-criterion task was continuous (ranging from -90° to 90°), we conducted a logistic regression on the covert-criterion data and a linear regression on the overt-criterion data.

These regressions provide a beta weight for each of the nine trials and provide insight into how the task is performed. For example, if the current trial’s decision in the covert-task was based on the difference between the current stimulus orientation and the average of orientations of the immediately preceding stimuli in each category (i.e., the limited-memory model), we would expect to find a positive weight for the current trial, negative weights with half the magnitude for the previous trial in each category, and zero weights for all other preceding trials. Alternatively, if the decision is based on the difference between the current stimulus and a weighted average of the past stimuli (i.e., the exponentially weighted moving-average model), we would expect to find a positive weight for the current stimulus and smaller negative weights for the previous trials with magnitudes that exponentially increase up to the preceding trial. The sign change between lag zero and lag one is consistent with previous findings [32]. Similarly, in the overt-criterion task, the beta weight for each of the nine trials provides insight into how the current criterion is set. For example, if the current criterion is set between the category means and each category mean is estimated by taking a weighted average of past stimuli from that category, we would expect to find positive beta weights that exponentially increase up to the preceding trial. That is, the criterion rotates in the same direction (clockwise or counter-clockwise) as the preceding ellipses’ orientations, regardless of the category, with more weight given to the most recently experienced ellipse.

#### Cross-correlation analysis

To determine how well observers tracked the changing category means in Expt. 2, in the overt-criterion experiment we computed the cross-correlation between the ideal criterion for an omniscient observer who knows the two category means on each trial (i.e., halfway between the true underlying category means for each trial) and each observer’s trial-by-trial criterion placement. The trial lag resulting in peak correlation provided an estimate of how quickly each observer updated the criterion.

#### Model fits

To obtain a quantitative measure of model fit, we computed Deviance Information Criterion (DIC) scores using Markov Chain Monte Carlo (MCMC) methods to sample model parameters from each potential model using Gibbs sampling [33] as implemented in JAGS (http://mcmc-jags.sourceforge.net/). DIC scores provide a measure for how well each model fits the data while penalizing for model complexity (effective number of parameters). DIC is a hierarchical modeling generalization of Akaike's Information Criterion (AIC). DIC is particularly useful when the posterior distributions of the models must be approximated using MCMC. A lower DIC score indicates a better model fit. Models with a DIC score 7 or more above that of the best-fitting model are considered poor models for the data [34,35,36].

For each observer, model and task, JAGS sampled the posterior of the model for 1000 adaptation steps, 1000 burn-in samples and 10,000 effective samples. Traces (i.e., the sequence of sampled values) for sensory uncertainty (*σ*_*v*_) in the covert-criterion task and adjustment uncertainty (*σ*_*a*_) in the overt-criterion task, deviance, and all other free parameters were monitored and estimates of the posterior density for each were calculated. Uninformative priors were used for all monitored parameters; these distributions were Jeffreys priors for all *σ* parameters (proportional to 1/*σ*) and flat distributions for all other parameters. Parameter lists for each model are shown in Tables 9 and 10. It is important to note that sensory uncertainty was not fit to the overt-criterion data but was fixed and set to each observer’s measured sensory noise. Thus, for the overt condition only adjustment uncertainty (*σ*_*a*_) was fit. Three chains were run, and visually checked for convergence for each parameter. Additionally, we report Gelman and Rubin's [37] potential scale reduction factor $\hat{R}$ for all parameters. Large values of $\hat{R}$ indicate poor convergence and values near one suggest convergence. The average value of $\hat{R}$ (across parameters and observers) was 1.0007 and all values were <1.1, indicating good convergence. Due to the possibility that observers’ data were generated using different models, we used random-effects Bayesian model selection for analysis at the group level [38]. This method is particularly useful when populations are heterogeneous and is more robust in the face of outliers than frequentist statistical tests (e.g., *t*-tests). Specifically, we used all subject-specific model evidence to compute the exceedance probability (i.e., the certainty with which we can conclude that model *k* is more likely than any other model, given the group data) for each model. The log evidence for each subject *i* and model *k* was approximated by −*DIC*_*i*,*k*_/2. This was computed for each task and condition. The group analysis was conducted using the open-source software package Statistical Parametric Mapping (SPM12; http://www.fil.ion.ucl.ac.uk/spm).

**Table 9:** Model parameters for the covert-criterion task.

Model	Number of parameters	Parameters
Ideal Bayesian	2	*σ*_*v*,_*σ*
Bayesian model selection—Expt. 1	1	*σ*_*v*_
Exponentially weighted moving-average	2	*σ*_*v*,_*α*
Reinforcement learning	2	*σ*_*v*,_*β*
Limited memory	1	*σ*_*v*_

**Table 10:** Model parameters for the overt-criterion task.

Model	Number of parameters	Parameters
Ideal Bayesian	2	*σ*_*a*,_*σ*
Bayesian model selection—Expt. 1	1	*σ*_*a*_
Exponentially weighted moving-average	2	*σ*_*a*,_*α*
Reinforcement learning	2	*σ*_*a*,_*β*
Limited memory	1	*σ*_*a*_

Note: Sensory uncertainty is not fit in the overt-criterion case because it was fixed in our models and set to an observer’s measured sensory uncertainty.

## References

[1] Green D.M., Swets J.A. (1966). Signal detection theory and psychophysics. New York: Wiley.

[2] Ackermann J.F., Landy M.S. (2015). Suboptimal decision criteria are predictd by subjectively weighted probabilities and rewards. Atten Percept Psychophys 77, 638–658. 10.3758/s13414-014-0779-z 25366822 PMC4336614

[3] Healy A.F., Kubovy M. (1981). Probability matching and the formation of conservative decision rules in a numerical analog of signal detection. J Exp Psychol Hum Learn 7, 344–354.

[4] Maddox W.T. (2002). Toward a unified theory of decision criterion learning in perceptual categorization. J Exp Anal Behav 78, 567–595. 10.1901/jeab.2002.78-567 12507020 PMC1284916

[5] Tanner W.P., Swets J.A., Green D.M. (1956). Some general properties of the hearing mechanism. Technical Report 30, Electronic Defense Group: University of Michigan.

[6] Tanner W.P.. (1956). Theory of recognition. J Acoust Soc Am 28, 882–888.

[7] Ulehla Z.J., (1966). Optimality of perceptual decision criteria. J Exp Psychol 71, 564–569. 5909083 10.1037/h0023007

[8] Akaishi R., Umeda K., Nagase A., Sakai K. (2014). Autonomous mechanism of internal choice estimate underlies decision inertia. Neuron 81, 195–206. 10.1016/j.neuron.2013.10.018 24333055

[9] Carandini M., Silva L., Busse L., Dakin S.C. (2012). Vision and superstition in mouse and man. J Vis 12(9):620.

[10] Fründ I., Wichmann F.A, Macke J.H. (2014). Quantifying the effect of intertrial dependence on perceptual decisions. J Vis 14(7):9, 1–16. 10.1167/14.7.9 24944238

[11] Yeshurun Y., Carrasco M., Maloney L.T. (2008). Bias and sensitivity in two-interval forced choice procedures: Tests of the difference model. Vision Res 48, 1837–1851. 10.1016/j.visres.2008.05.008 18585750 PMC5839130

[12] Yu A.J., Cohen J.D. (2009). Sequential effects: Supersition or rational behavior? Advances in Neural Information Processing Systems 21, 1873–1880. PMC4580342 26412953

[13] Lages M., Treisman M. (2010). A criterion setting theory of discrimination learning that accounts for anisotropies and context effects. Seeing Perceiving 23, 401–434. 21466134 10.1163/187847510x541117

[14] Fründ I., Haenel N.V., Wichmann F.A. (2011). Inference for psychometric functions in the presence of nonstationary behavior. Journal of Vision 11(6):16, 1–19. 10.1167/11.6.16 21606382

[15] Burge J., Ernst M.O., Banks M.S. (2008). The statistical determinants of adaptation rate in human reaching. J Vis 8(4):20, 1–19. 10.1167/8.4.20 PMC2684526 18484859

[16] Landy M.S., Goutcher R., Trommershäuser J., Mamassian P. (2007). Visual estimation under risk. J Vis 7(6):4, 1–15. 10.1167/7.6.4 PMC2638507 17685787

[17] Landy M.S., Trommershauser J., Daw N.D. (2012). Dynamic estimation of task-relevant variance in movement under risk. J Neurosci 32, 12702–12711. 10.1523/JNEUROSCI.6160-11.2012 22972994 PMC3477850

[18] Qamar A.T., Cotton R.J., George R.G., Beck J.M., Prezhdo E., et al (2013). Trial-to-trial, uncertainty-based adjustment of decision boundaries in visual categorization. Proc Natl Acad Sci USA 110, 20332–20337. 10.1073/pnas.1219756110 24272938 PMC3864339

[19] Summerfield C., Behrens T.E., Koechlin E. (2011). Perceptual classification in a rapidly changing environment. Neuron 71, 725–736. 10.1016/j.neuron.2011.06.022 21867887 PMC3975575

[20] Trommershäuser J., Gepshtein S., Maloney L.T., Landy M.S., Banks M.S. (2005). Optimal compensation for changes in task-relevant movement variability. J Neurosci 25, 7169–7178. 10.1523/JNEUROSCI.1906-05.2005 16079399 PMC6725228

[21] Daw N.D., O'Doherty J.P., Dayan P., Seymour B., Dolan R.J. (2006). Cortical substrates for exploratory decisions in humans. Nature 441, 876–879. 10.1038/nature04766 16778890 PMC2635947

[22] Beck J.M., Ma W.J., Pitkow X., Latham P.E., Pouget A. (2012). Not noisy, just wrong: The role of suboptimal inference in behavioral variability. Neuron 74, 30–39. 10.1016/j.neuron.2012.03.016 22500627 PMC4486264

[23] Acuña D.E., Schrater P. (2010). Structure learning in human sequential decision-making. PLoS Comput Biol 6(12):e1001003. 21151963 10.1371/journal.pcbi.1001003 PMC2996460

[24] Chen S.Y., Ross B.H., Murphy G.L. (2014). Implicit and explicit processes in category-based induction: is induction best when we don't think? J Exp Psychol Gen 143, 227–246. 10.1037/a0032064 23506087

[25] Trommershauser J., Maloney L.T., Landy M.S. (2008). Decision making, movement planning and statistical decision theory. Trends Cogn Sci 12, 291–297. 10.1016/j.tics.2008.04.010 18614390 PMC2678412

[26] Hertwig R., Barron G., Weber E.U., Erev I. (2004). Decisions from experience and the effect of rare events in risky choice. Psychol Sci 15, 534–539. 10.1111/j.0956-7976.2004.00715.x 15270998

[27] Brainard D.H. (1997). The Psychophysics Toolbox. Spat Vis 10, 433–436. 9176952

[28] Pelli D.G. (1997). The VideoToolbox software for visual psychophysics: transforming numbers into movies. Spat Vis 10, 437–442. 9176953

[29] Levitt H. (1971). Transformed up-down methods in psychoacoustics. J Acoust Soc Am 49, 467–477. 5541744

[30] Anderson B.D.O., Moore J.B. (1979). Optimal Filtering. Englewood Cliffs, NJ: Prentice-Hall.

[31] Wichmann F.A., Hill N.J. (2001). The psychometric function: I. Fitting, sampling, and goodness of fit. Percept Psychophys 63, 1293–1313. 11800458 10.3758/bf03194544

[32] Bayer H., Glimcher P.W. (2005). Midbrain dopamine neurons encode a quantitative reward prediction error signal. Neuron 47, 129–141. 10.1016/j.neuron.2005.05.020 15996553 PMC1564381

[33] Gelman A., Carlin J.B., Stern H.S., Dunson D.B., Vehtari A., et al (2014). Bayesian Data Analysis. New York: CRC Press.

[34] Burnham K.P., Anderson D.R. (1998). Model Selection and Inference. New York: Springer.

[35] Spiegelhalter D.J., Best N.G., Carlin B.R., van der Linde A. (2002). Bayesian measures of model complexity and fit. J R Stat Soc Series B Stat Methodol 64, 583–616.

[36] Kass R.E., Raftery A.E. (1995). Bayes Factors. J Am Stat Assoc 90, 773–795.

[37] Gelman A., Rubin D.B. (1992). Inference from iterative simulation using multiple sequences. Stat Sci 7, 457–472.

[38] Stephan K.E., Penny W.D., Daunizeau J., Moran R.J., Friston K.J. (2009). Bayesian model selection for group studies. NeuroImage 46, 1004–1017. 10.1016/j.neuroimage.2009.03.025 19306932 PMC2703732

